# Mind Cure, No. 2

**Published:** 1888-06

**Authors:** 


### MIND CURE NO. 2.

### COURSE OF STUDY AS TAUGHT BY MRS. NEWMAN, OF BOSTON.

I.

God is mind; there is nothing but mind and expression of mind; from man himself to a simple child's toy, the only eternal reality is eternal, immortal mind. Mind manifests itself by and through and by means of ideas; it is impossible to conceive of a mind without thoughts. or thoughts without a mind—they naturally imply each other. A mind is not its thoughts, and all thoughts combined are not the mind that thinks them. We all exist as ideas in infinite mind. As the artist holds the ideal picture in mind, so also God holds the ideal man; we have our real existence in the mind of God. Our ideality is an expression of God's mind. My individuality, my body, is an expression of my mind.

Ideas are the living causes of things. All causes are absolutely invisible to the external sense. The kingdom of God is within ; health is within. The true cause of health and happiness is within our own spirit awaiting recognition. Mind is the natural stimulus of the body ; muscular power is mental power.

Our rules must be studied and committed to the life in a spirit of repose, and it must be in a spirit of repose that we practice self cure, and in self meditation there must be no haste.

What we struggle and hope for is accomplished ; the power of maintaining the true life against the collective falsities, depends upon energy. There are several kinds of energy ; do not mistake self-will for energy

Self knowledge must be sought. It is only by cheerfulness that we can preserve health. Sadness springs from evil ; comfort and despair lie within us ; what the spirit wills the body must. You cannot at one and the same time think yourself both ill and well. You can think what you please ; but you never can be well until you cease to think yourself ill ; pronounce yourself in health. The mind can sow the seed for the destruction or preservation of the body. Mind penetrates all matter ; nothing can resist it.

Fear is a state of weakness during which you are easily conquered by an enemy ; we are not disturbed by things that happen, but by our fears about the things. Death is not terrible in reality ; it is only the opinion that we have of death.

II.

God and man cannot be separated. God is infinite mind ; a mind without thoughts is unthinkable.

Thoughts have no undivided existence of their own ; they cannot exist independent of mind. Thoughts are not the mind that thinks, neither is the mind the thoughts, but neither can exist without the other. They mutually imply each other—they cannot exist apart.

God is mind ; we all exist as ideas in the mind of God ; nothing but perfection comes from perfection. The ideal man in the perfect infinite mind must be perfect. That the body expresses imperfection is only that the mind of the individual holds imperfect thoughts. Thought is the source of all there is. If the thought of God does not cause and pervade all things, it does not exist. God is life ; disease and death are not in life, consequently they do not exist. Existence is from God. We exist in God. Existence is the projecting, the going forth from anything, and God has existence through man.

Man the spiritual, is a direct outcome of the divine mind, and has his being in life, harmony, justice, love and power ; the material body, as we now see it, is a projection or outbirth from the mortal mind. Mortal mind is merely a collection of false thoughts held regarding man, God and the visible universe. Our body has no independent existence ; our body has only a seeming life ; spiritual essences are the only real things in the universe.

Thoughts are patterns or models from which all things appear. Our bodies as we see them in space are distorted, imperfect imitations of the divine man made after God's own image ; our identity is the idea held in the infinite mind. Our personality is the ego of the personal man, the individual separated from the universal by feelings; consciousness ; this consciousness is spiritual consciousness and only recognizes spirit ; this personality is a reflection or shadowing forth of the identity or idea held in the mind of God—the outer man, (not the body, for that is only an expression of the thoughts, the life of the outer man) is only a reflection of a reflection, an inverted image of ourselves ; man perceives himself in direct contradiction to the truth.

The following are the traits working forth as diseases on the body : selfishness, greed, pride, egotism, vanity, obstinacy, hatred, malice, envy, fear, indolence, intemperance, hypocrisy, censoriousness, backbiting, sensuality, skepticism, grief, sorrow, love of creeds, love of rule, impatience, restlessness, discontent, diffidence, quick temper, distrust, fear of death, fear of fire, of falling, fear of pain, of poverty, disgrace, of hell fire, fear of annihilation, of people's opinions, home-sickness, shame, disgust, anxiety, self-depreciation, gloom, ambition, fault-finding, fickleness, uncertainty, love of opposition, fear of being buried alive, fear of accident, sensitiveness.

III.

Do not put on the thoughts of others, but think them out for yourself. You must not hope or expect, but you must know with confidence. Do not try to conquer disease. A thought of conflict gives power to evil. Why should we fight againt a thing that has no power ? When you see the true value of evil and disease you will despise them. Changing from natural to spiritual thought, from the idea of disease to the idea of health, is like going into another room and closing the door. When we know a shadow to be a shadow it has no power over us. We each have a sphere about us, a sphere of thought and motive that effects every one with whom we come in contact.

You grow faster by mistakes than by a succession of successes. Every evil and false thought weighted with the strength of your own belief goes out to add its weight to every passive mind. Every true thought helps raise humanity and lets the light in. One false thought adds to the race belief in the power of evil. Watch your own thoughts ; bring every thought into subjection to truth. Deny your falsities or beliefs in disease of body or mind by trusting in their opposites which are real, and you will prove that your sufferings were shadows. Deny all mortal inheritance. You inherit only from your source. Can you inherit rheumatism from spirit ? God is nearer to you than the atmosphere of your natural body.

There are three fears to be conquered when you attempt to treat anyone : one the fear you have in your own mind, the fear the patient has and the fear in the medical world.

It is much better to treat the patient when asleep than when awake, except at a distance ; when treating at a distance blot out all thoughts of distance.

Whenever it comes to you to fear disease, or pain, or accident, say to yourself, "I am spirit ; I have my existence not in space, but in the mind of God ; mind is my environment, and nothing can harm me ; I am a manifestation of immortal spirit, and am myself immortal ; I cannot fear death, for I know there is no death ; I am held in pirit ; spirit is life and you cannot escape life ; it is as impossible to go from life as it is to go from God, for God is life. I am alive and always will be ; I cannot fear pain for spirit cannot comprehend pain."

IV.

Life cannot know death ; light cannot know darkness ; spirit is truth, it cannot think falsely, and I know that pain is a false belief. I cannot fear an accident, for the spirit cannot be touched by a material body ; matter offers no resistence to spirit ; I am spirit and rest in safety ; I do not defy harm, for evil has no power to defy. I live and have my being in mind, spirit, life, light, harmony, and truth, and for me the opposites, the contradictions of these have no existence.

God is mind ; I exist in that mind and partake of its qualities. God is infinite life. Substance is that which stands under the foundation. God is infinite life, substance, and intelligence, and he bestows upon us all of his life substance and intelligence that we will receive. We must become transparent lenses for the light to shine through, unobstructed and unchanged. When we hold our minds contracted, distorted or fixed in a false idea, the life or light coming through their mental forms seems to be changed as does the light of the sun in shining through a different colored glass. Strive after innocence, not the innocence of childhood; innocence is a step of reception, an intelligence to receive all from God; not the utterance of ignorance, but the innocence of wisdom.

We say there is nothin but infinite life, substance, and intelligence. Life never results in death. Infinite intelligence never reported death or forms of disease; therefore if life never created, substance never held, or intelligence never reported death or forms of disease, they cannot be in existence. I never can suffer from them, for I, being a manifestation of this life, a partaker of this substance and intelligence, can only have that which comes from this life as contained in this substance, and is reported by this intelligence.

V.

Infinite, infinity—not finite—signifies that which is limitless, without boundary, unbounded. As mind is the governing principle of the body, so iufinite mind, boundless, is the governing power of the universe. Man's identity exists in infinite mind as an idea—the ideal mind partakes of the qualities that hold it—is immortal, harmonious, alive, for infinite mind is life itself. This infinite mind—God—is nearer to us than the atmosphere to our natural bodies, and we can not escape it; death to us is an impossibility.

Do not give up to the temptation to think over your ills; never while under treatment from your own mind or others think over your own case to decide whether you are improving. Forget yoor old self; become so fixed in the new habit that the old has passed from your memory. Become positive and reject all forms of evil; fear is an insult to the creator; can you imagine a child continually crying for fear that the parents will forget to feed and clothe it? We must become as little children in our perfect trust; the child knows enough to remain in its father's home. We not only leave our father's house but wander into a foreign country. Have perfect confidence in the spirit within you. The unconscious mind is filled with a man's love,—ruling desires; it represents the man's habit of thought.

Keep your eye fixed upon the copy, the ideal man in the mind of God; and your whole being, even to the outermost, shall be regenerated and recreated after that perfect model.

